# Structural characterization of recombinant human CD81 produced in *Pichia pastoris*

**DOI:** 10.1016/j.pep.2007.10.013

**Published:** 2008-02

**Authors:** Mohammed Jamshad, Sundaresan Rajesh, Zania Stamataki, Jane A. McKeating, Timothy Dafforn, Michael Overduin, Roslyn M. Bill

**Affiliations:** a School of Life and Health Sciences, Aston University, Aston Triangle, Birmingham B4 7ET, United Kingdom; b CRUK Institute for Cancer Studies, The University of Birmingham, Edgbaston, Birmingham B15 2TT, United Kingdom; c Division of Immunity and Infection, Institute of Biomedical Research, Medical School, University of Birmingham, Birmingham B15 2TT, United Kingdom; d Department of Biosciences, University of Birmingham, Birmingham B15 2TT, United Kingdom

**Keywords:** Membrane proteins, Protein production, Yeast, Circular dichroism, Analytical ultracentrifugation

## Abstract

Human CD81 (hCD81) protein has been recombinantly produced in the methylotrophic yeast *Pichia pastoris*. The purified protein, produced at a yield of 1.75 mg/L of culture, was shown to interact with Hepatitis C virus E2 glycoprotein. Immunofluorescent and flow cytometric staining of *P. pastoris* protoplasts with monoclonal antibodies specific for the second extracellular loop (EC2) of hCD81confirmed the antigenicity of the recombinant molecule. Full-length hCD81 was solubilized with an array of detergents and subsequently characterized using circular dichroism (CD) and analytical ultracentrifugation. These biophysical techniques confirmed that the protein solution comprises a homogenous species possessing a highly-defined α-helical secondary structure. The predicted α-helical content of the protein from CD analysis (77.1%) fits remarkably well with what would be expected (75.2%) from knowledge of the protein sequence together with the data from the crystal structure of the second extracellular loop. This study represents the first biophysical characterization of a full-length recombinant tetraspanin, and opens the way for structure–activity analyses of this ubiquitous family of transmembrane proteins.

CD81 is a four transmembrane domain membrane protein of the tetraspanin (TSP) superfamily [1]. To date 34 family members have been identified in mammals, of which 33 are present in humans. Common to all tetraspanins are the four transmembrane (TM) spanning regions containing several conserved polar residues and, in most cases, relatively short cytoplasmic amino- and carboxy-terminal tails [2]. These TM domains are linked via a short extracellular loop 1 (EC1)^2^ and a larger extracellular loop 2 (EC2) containing the highly conserved Cys-Cys-Gly motif unique to tetraspanins. Between 4 and 8 Cys residues can be found in the EC2 loop. These residues form several disulfide bridges [3], which are critical for correct folding of the EC2 domain and subsequent interaction with ligands and partner proteins [2,4]. In human CD81 (hCD81) there are four such residues (Fig. 1).

The exact biochemical function of tetraspanins is not well understood, but they are implicated in a variety of key cellular processes including oocyte fertilization, tissue differentiation, cell adhesion, tumor growth and cell metastasis [4]. Emerging evidence also reveals tetraspanin association with signaling lipids, cytoplasmic proteins [4] and adhesion receptors of the integrin family to facilitate the assembly of tetraspanin-enriched microdomains also known as the ‘tetraspanin web’. In the case of hCD81, it has a role in B- and T-cell activation [4,5] and interacts with Ig superfamily members, CD4, CD8, CD19 and EWI-proteins [6]. Recent research has also revealed that hepatitis C virus (HCV) E2 envelope glycoprotein binds to the EC2 domain of hCD81 [7] and that hCD81 is a critical co-receptor defining HCV entry [8–10]. hCD81 is further implicated in *Plasmodium falciparum* infectivity, leading to malaria [11].

Despite their intriguing roles in so many normal and disease states, tetraspanin structure–activity relationships are poorly understood. To date the only crystal structure of any tetraspanin is that of the soluble EC2 domain of hCD81 [3], which reveals a mushroom shaped loop confirming the presence of the highly conserved Cys-Cys-Gly motif and two intact disulfide bridges. However, elucidation of the full-length hCD81 structure is necessary in order to facilitate an understanding of *in vivo* binding and the role of the TM domains in specific interactions with biologically-relevant ligands. As a consequence, structural information of a full-length tetraspanin would provide valuable insight into possible mechanisms of action. Moreover, since about 2% of the world’s population is chronically infected with HCV, resulting in hepatitis, cirrhosis, liver failure and hepatocellular carcinoma, hCD81 is a clinically-important anti-viral drug target.

Tetraspanins (like other human membrane proteins) have been difficult to overproduce in a purified form for detailed biophysical analyses. In this study we report the production of hCD81 in *Pichia pastoris* as well as its optimized solubilization and purification which yields milligram quantities of correctly-folded, pure protein for biophysical characterization. Structural integrity was followed throughout production via binding of conformationally-specific anti-hCD81 monoclonal antibodies [12] to both membrane-integrated and detergent-solubilized hCD81. Function was confirmed by binding to a known ligand, HCV E2 glycoprotein [7]. Using circular dichroism (CD) and analytical ultracentrifugation (AUC), the purified protein was shown to be recovered as a highly-pure, homogeneous species that is predominantly α-helical in nature. This study represents the first biophysical characterization of a recombinant tetraspanin, and paves the way for detailed structure–function analyses of tetraspanins using NMR and X-ray crystallography.

## Materials and methods

### Plasmid construction

**Fig. 1:**
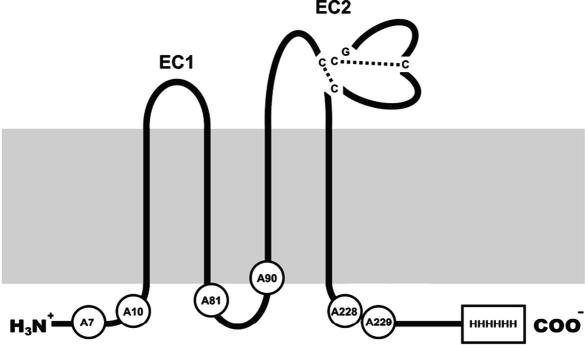
Schematic representation of the hCD81 protein produced in this study. The mutated palmitoylation sites (Cys to Ala) are highlighted as white circles in the sequence. The His_6_ tag is indicated as a rectangle in the carboxy-terminal tail. The two extracellular loops are marked as EC1 and EC2. EC2 contains the conserved Cys-Cys-Gly (CCG) motif associated with the tetraspanin family. The published crystal structure of soluble EC2 indicates the importance of this motif in forming the disulfide bridges represented in the figure. The membrane is represented in grey.

DNA encoding hCD81 (GeneID: 975) was amplified using a 3-step, site-specific mutagenesis PCR approach to remove all palmitoylation sites (Cys to Ala) in the central segment of hCD81 as indicated in the protein sequence (Fig. 1). The first hCD81 amplification product incorporated an *Eco*RI site (underlined) followed by a Kozak consensus sequence (bold) and removed the palmitoylation sites in the native sequence through the use of the forward primer 1, 5′-GGTCGGAATTC**AAAATGTCT**GGAGTGGAGGGCGCCACCAAGGCCATCAAGTACCTG-3′ and reverse primer 1, 5′-GGATTCCTGGATGGCCCCGTAGGCGCCCAGGAAGCCAACGAACA T-3′. Forward primer 2, 5′-TACGGGGCCATCCAGGAATCCCAGGCTCTGCTGGGGGACGTT-3′ (overlapping reverse primer 1) in combination with reverse primer 2, 5′-GCACCCTCGAGTCA**ATGATGATGATGATGATG**GCCGCCGCCGTACACGGAGCTGTTCCGGATGCCTGCGGCCAGCACCAT-3′ was used to remove all remaining palmitoylation sites and introduce a carboxy-terminal His_6_ tag (bold), a stop codon and a *Xho*I site (underlined). The full-length, mutant product was amplified using forward primer 1 and reverse primer 2 and cloned into the *P. pastoris* vector pPICZB using the *Eco*RI and *Xho*I sites.

### Yeast strains, growth conditions and expression screening

**Fig. 2:**
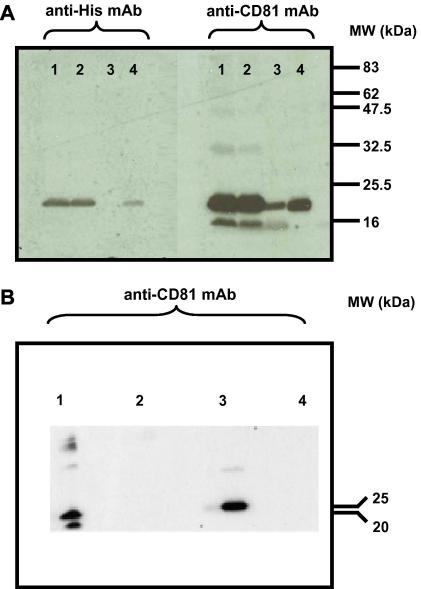
Immunoblot of hCD81 isolated from *P. pastoris* membranes expressing pPICZB-hCD81-His_6_. hCD81 was identified using both a commercial monoclonal anti-His_6_ antibody (Clontech; left panel) and an anti-hCD81 monoclonal antibody (right panel). Lane 1: total cell lysate, lane 2: supernatant following centrifugation at 10,000*g* for 30 min, lane 3: supernatant after ultracentrifugation at 100,000*g* for 90 min, lane 4: total membrane fraction. Samples were not exposed to any reducing agents. Positions of molecular mass markers (kDa) are indicated on the right-hand side of the figure (A). The conformational specificity of the anti-hCD81 monoclonal antibody was confirmed by probing reduced and non-reduced hCD81 samples. Lane 1: MDA-MB-231 cell-line (non-reduced), lane 2: MDA-MB-231 cell-line (reduced with 1% β-mercaptoethanol), lane 3: total membrane fraction from *P. pastoris* membranes expressing pPICZB-hCD81-His_6_ (non-reduced), lane 4: total membrane fraction from *P. pastoris* membranes expressing pPICZB-hCD81-His_6_ (reduced with 1% β-mercaptoethanol). Positions of molecular mass markers (kDa) are indicated on the right-hand side of the figure (B).

The hCD81 expression plasmid was linearized with the restriction endonuclease *Pme*I, purified, concentrated to 0.5–1 μg DNA/μL, and used to transform *P. pastoris* wild-type strains X-33 and GS115 (Invitrogen) by electroporation, as described by the manufacturer (Invitrogen) using *P. pastoris* competent cells produced as described by Cereghino and colleagues [13]. Ten transformants were cultured in BMGY medium (1% yeast extract, 2% peptone, 1.34% yeast nitrogen base without amino acids, 0.00004% biotin, 1% glycerol, 0.1 M phosphate buffer, pH 6) at 30 °C and 230 rpm overnight to yield an OD_600_ of 2–10. Production screening for hCD81 was induced in 3 mL BMMY medium (BMGY containing 1% methanol instead of 1% glycerol) at 30 °C and an initial OD_600_ of 1 in 24-well uniplates (Whatman). Protein production was maintained by addition of methanol (to a final concentration of 1% (v/v)) 24 h and 48 h post-induction. Samples were collected by centrifugation at 6, 24 and 54 h post-induction to analyze production yields and determine the optimal harvest time. Supernatants were decanted and pellets were then frozen in liquid N_2_, and stored at −80 °C. For reducing SDS–PAGE, whole cell lysates from each time point were prepared by heating these cell pellets at 98 °C for 10 min in sample buffer (50% distilled water, 12.5% 0.5 M Tris–HCl, pH 6.8, 10% glycerol, 2% SDS, 5% β-mercaptoethanol and 0.001% (w/v) bromophenol blue). They were then loaded onto a 12% Tris–HCl gel, transferred to a nitrocellulose membrane (Hybond ECL, GE Healthcare), and analyzed by immunoblotting with either a primary monoclonal anti-His_6_ antibody (Clontech) or a primary anti-hCD81 monoclonal antibody together with an anti-mouse IgG HRP-conjugated secondary antibody (Sigma). For non-reducing SDS–PAGE, β-mercaptoethanol was omitted from the sample buffer. The samples in Figs. 2 and 4 are loaded in SDS–PAGE loading buffer without the addition of β-mercaptoethanol. This means that the proteins have been partially denatured with SDS, but not reduced. In Fig. 5, the SDS–PAGE loading buffer contains β-mercaptoethanol, meaning that the proteins are fully denatured. The protein signal was detected by EZ-ECL chemiluminescence (Geneflow) and analyzed with a UVIprochemi imaging system (UVItec). The highest yielding *P. pastoris* transformant (subsequently referred to as X33-hCD81) identified by this analysis was cultured in 10 mL of BMGY medium overnight at 30 °C at 230 rpm. This culture was subsequently inoculated to OD_600_ = 1 into a sterile 250 mL shake flask containing 50 mL of BMMY. The culture was supplemented with methanol (final concentration of 1% (v/v)) 24 and 48 h post-induction. After 54 h, the culture was centrifuged and the pellet was retained for protein analysis and purification. For large-scale protein production and purification, *P. pastoris* wild-type strain X-33 producing recombinant hCD81 was grown in 2 L of BMMY, inoculated to a final OD_600_ = 1 in a 5 L shake flask for 54 h with the addition of methanol (final concentration of 1% (v/v)) 24 h and 48 h post-induction.

### Membrane preparation

All work was carried out at 0–4 °C. Twenty grams of X33-hCD81 cells were suspended in 40 mL ice-cold breaking buffer (50 mM sodium–phosphate buffer, pH 7.4, 100 mM NaCl, 5% glycerol, 2 mM PMSF) and poured into an Avestin-C3 cell disrupter. Yeast cells were broken by three passages through the chilled cell, and breaking efficiency was >90% when inspected with a light microscope. Unbroken cells and cell debris were removed from the membrane suspension by low-speed centrifugation (10,000*g*, 30 min, 4 °C). Membranes were then collected using an ultracentrifuge (100,000*g*, 90 min, 4 °C), suspended in solubilization buffer (20 mM Hepes, pH 7.4, 100 mM NaCl, 10% glycerol, 1 mM PMSF). The protein content of the membrane fraction was quantified using the Bio-Rad Protein Assay Kit [14]. Standard curves were prepared each time the assay was performed as described by the manufacturer, using aqueous solutions of bovine serum albumin (Fraction V) as a standard. Membranes were snap-frozen in liquid nitrogen and stored at −80 °C. Immunoblots on membrane preparations were performed as described above.

### Solubilization and purification

A detergent screen was performed on hCD81-containing membranes (with a total protein content of 0.6 mg protein) in 100 μL solubilization buffer (20 mM NaPO_4_, pH 7.4, 250 mM NaCl, 10% glycerol, 100 μM 4-(2-aminoethyl)benzenesulphonyl fluoride (Roche), AEBSF) using the following panel of detergents at 2% w/v on membrane suspensions: *n*-dodecyl-β-d-maltopyranoside (DDM), *n*-dodecylphosphocholine (DPC), cyclofos-4 (CYFOS-4), *n*-octyl-β-d-glucopyranoside (β-OG), foscholineiso9 (FCI09), lauryldimethylamine oxide (LDAO), pentaethyleneglycol-*n*-octylether (C8E5), *n*-dodecylphosphocholine–cholesterolhemisuccinate (DPC/CHS) and docosaethyleneglycol–monohexadecylether (Brij 58; all purchased from Anatrace Inc). Samples were incubated for 1 h at room temperature with mild agitation. Non-solubilized material was removed by centrifugation at 18,000*g* for 2 h at 4 °C. The solubilized (supernatant) and non-solubilized (membrane pellet) material were analyzed by immunoblot analysis for solubilization efficiency using a primary anti-hCD81 monoclonal antibody and an anti-mouse IgG HRP-conjugated secondary antibody (Sigma). For scale-up, 10 mL of membranes (300 mg protein) were added to 10 mL of solubilization buffer containing detergent to a final working concentration of 5%. The suspension was incubated at room temperature for 2 h on a rotary mixer. Non-solubilized material was removed by centrifugation at 100,000*g* for 1 h at 4 °C. The supernatant was then diluted to 2.5% detergent by adding 20 mL of solubilization buffer to a final concentration of 20 mM imidazole and then applied onto a 1 mL HisTrap HP column (GE Healthcare) pre-equilibrated with 2.5% detergent plus 20 mM imidazole in solubilization buffer. The column was washed with two column volumes of the same buffer followed by a further three column volumes of 1% β-OG-containing buffer. The hCD81 fractions were eluted with a linear gradient of 20–500 mM imidazole in solubilization buffer containing 1.0% β-OG. The eluted fractions were subsequently concentrated and analyzed by SDS–PAGE and immunoblotting. Electrophoresis was carried out using an X-Cell™ Mini-Cell System (Novex, San Diego, USA) at 200 V for 45 min. Proteins were visualized using Coomassie Brilliant Blue (0.1% w/v) in an aqueous solution of 40% methanol (v/v) and 10% glacial acetic acid (v/v) for 60 min and then destained in an aqueous solution of 40% (v/v) methanol with 10% (v/v) glacial acetic acid.

### Protoplast production and analysis

A mid-log phase aliquot of *P. pastoris* X33-hCD81 cells was resuspended in 50 mM phosphate buffer, pH 7.4 containing 0.5 mM MgCl_2_ at 1 × 10^6^ cells/mL. Cells were collected by centrifugation at 500*g* for 3 min and suspended in 1 mL of the same buffer containing 3.7% paraformaldehyde (PFA) to fix the cells, and incubated at room temperature for 1 h. The cells were washed twice with PFA-free buffer and once in buffer containing 1.2 M sorbitol (sorbitol buffer). Cells were finally suspended in 0.5 mL sorbitol buffer and treated with zymolyase by adding 50 μL of 10 mg/mL zymolyase-20T and incubating at 37 °C for 1 h. After washing cells once and then suspending them in sorbitol buffer, 6 μL of cells were added to a 0.1% poly-l-lysine-coated slide and incubated for 30 min to attach. Slides were gently washed with phosphate-buffered saline (PBS), permeabilized with 1% Triton X-100 in PBS, blocked and subsequently stained with primary and fluorochrome-conjugated secondary antibodies as previously described [15,16]. In addition, they were incubated in PBS containing 1% bovine serum albumin (BSA) in the presence or absence of 0.1% saponin and incubated with anti-hCD81 (M38) or a control isotype monoclonal antibody on ice for 1 h. Cells were washed in PBS containing 1% BSA in the presence or absence of 0.1% saponin and bound antibody detected by incubating the cells with anti-mouse alexa 488 (R&D Systems) for 1 h, washing to remove unbound antibody and analyzing by flow cytometry. For the surface staining, the intact protoplasts were stained first and fixed subsequently in 1% PFA before assaying fluorescence by flow cytometry.

### Biophysical analyses

A Beckman XLI analytical ultracentrifuge using an 8 cell 50Ti rotor was used for the AUC studies. Samples of hCD81 were prepared in 20 mM NaPO_4_, pH 7.4, 250 mM NaCl, 10% glycerol, 100 μM AEBSF (solubilization buffer) and 1.0% β-OG and were loaded into two sector cells and centrifuged at 40,000 rpm for 8 h at 4 °C. The absorbance of the sample was measured at a wavelength of 220 nm throughout the cell. A total of 100 measurements were taken throughout each 8 h run. Data from each experiment were analyzed using the continuous c(s) distribution model implemented within SEDFIT94 [17]. Parameters for the partial specific volume of the protein, buffer viscosity and density were calculated using SEDNTERP [18].

Circular dichroism spectra were recorded using a JASCO J-810 nitrogen-flushed spectropolarimeter. Samples of hCD81 were prepared in solubilization buffer plus 1.0% β-OG or DPC. Far-UV CD spectra of samples were collected using cell pathlengths of 0.2 cm in the far-UV domain and at 25 ± 0.1 °C for all experiments, unless otherwise stated. Spectra were acquired by averaging eight scans and corrected for buffer signal. Data deconvolution was carried out using CDSSTR [19] using the SMP50 basis set that contains 37 soluble and 13 membrane protein reference spectra.

### Molecular structure analysis

The X-ray crystal structure of the large extracellular loop (EC2) of hCD81 (PDB ID 1IV5) was analyzed using Swiss PDB [20]. Secondary structure content was calculated using the routines within Swiss PDB.

### Measurement of HCV E2–hCD81 interaction

Purified, full-length, recombinant hCD81 together with bacterially-produced hCD81 EC2 (the positive control; [21]) and bacteriorhodopsin (the negative control) were allowed to bind to microtiter plates (Costar High-bonding polystyrene, Corning Corp.) overnight at 4 °C. Plates were blocked with 1% BSA and washed with PBS containing 0.1% Tween 20 (blocking buffer). To confirm that hCD81 antigens had bound the plate, antigen was detected with anti-CD81 monoclonal antibody, M38 (1 μg/ml). After a 1 h incubation at 37 °C, plates were washed and bound antibody was visualized with a horseradish peroxidase (HRP)-conjugated anti-mouse antibody (Caltag Laboratories, Inc.) and tetramethyl benzidene (TMB) substrate. HCV strain H77 (genotype 1) soluble E2 was generated in human embryonal kidney cells as previously described [21] and added to hCD81 and control-antigen-coated plates, and incubated for 1 h at 37 °C. Bound E2 was detected with rat anti-E2 mAb, 9/27, and realized with HRP-conjugated anti-rat antibody (Caltag Laboratories, Inc.) and TMB substrate.

## Results

### hCD81 can be produced in *P. pastoris*

The entire hCD81 coding sequence was tagged at the 3′ end with a six histidine tag coding sequence and cloned into the *P. pastoris* expression vector pPICZB downstream of the alcohol oxidase 1 (*AOX1*) promoter. In order to prevent aggregation and thus to obtain homogeneous recombinant protein from *P*. *pastoris*, the palmitoylation sites were eliminated by conservative point mutations to give the sequence shown in Fig. 1. It has previously been shown that the removal of palmitoylation sites in various tetraspanins does not affect their overall conformation (unpublished data and [15]). Successful transformants were identified through small-scale (3 mL) expression screens. The highest levels of hCD81 expression were observed at 54 h post-induction in the wild-type X33 strain of *P. pastoris* (transformant X33-hCD81) in comparison to the GS115 strain (data not shown).

In order to generate large amounts of biomass for subsequent protein purification, biophysical and biochemical studies, flask cultivations (2 L) of transformant X33-hCD81 were harvested 54 h post-induction. Cellular localization and structural integrity of hCD81 within the membrane was established by immunoblot staining, yielding a positive immunoblot signal both in the total cell lysate and the membrane fraction (Fig. 2A). Fig. 2B confirms that only the non-reduced form of hCD81 (both from a hCD81-producing cell-line and from our recombinant membranes), in which the critical disulfide bridges of EC2 are intact, is recognized by the conformationally-specific antibody. The protein gels for these immunoblots depicted the expected ladder of bands (data not shown).

**Fig. 3:**
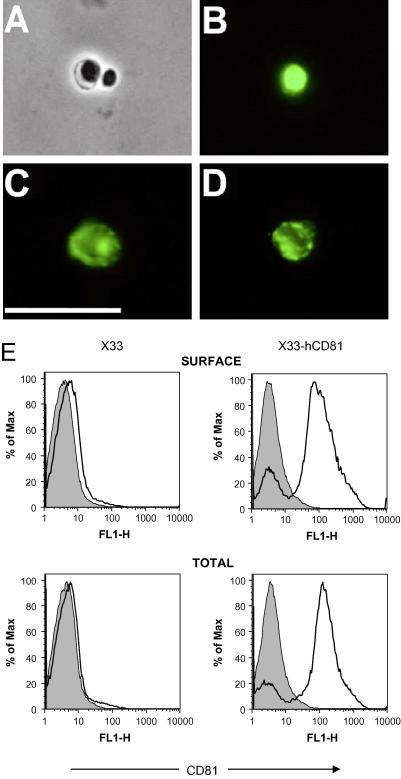
hCD81 produced in *P. pastoris* is recognized by monoclonal antibodies to the native protein. *P. pastoris* strains X33 and X33-hCD81 were fixed in paraformaldehyde and protoplasts were produced by zymolyase treatment. Permeabilized X33-hCD81 protoplasts (in Triton X-100) were stained with mAbs to hCD81: M38 (B), 1D6 (C) and mAb 5A6 (D). Staining was visualized with goat anti-mouse IgG Ab conjugated to alexa 488. Panel A shows a phase-contrast image of protoplasts shown in panel B. Note, that only one of the two protoplasts is stained with anti-hCD81 mAb. This may be due to intrinsic heterogeneity of the culture of *P. pastoris* expressing hCD81. The scale bar represents 10 μM. Panel E shows intact or saponin-permeabilized protoplasts which were incubated with anti-hCD81 mAb M38 (open histogram) and an isotype-matched control irrelevant IgG (grey histogram) for 1 h on ice. Bound mAb was visualized with anti-mouse Ig alexa 488 and analyzed by flow cytometry. Histograms represent the staining intensity of the bound antibodies.

Immunofluorescence studies showed that three anti-hCD81 monoclonal antibodies (mAb): M38, 5A6 and 1D6 [22–24] bound to hCD81-producing yeast protoplasts, suggesting that the recombinant protein is intact in the yeast membrane (Fig. 3A–D). In control experiments we detected no staining with anti-hCD81 mAbs in non-expressing *P. pastoris* X33 cells and thus observed only a black panel (data not shown). Furthermore, the mAb against tetraspanin hCD82 did not stain hCD81-expressing yeast protoplasts (data not shown). This confirmed the specificity of the anti-hCD81 antibody staining. Flow cytometric studies further showed that approximately 80% of the protoplasts had produced hCD81 and that this did not increase significantly after permeabilizing with saponin (Fig. 3E). This staining was specific as M38 failed to bind to X33 protoplasts and isotype-controlled mAb failed to bind to X33-hCD81 cells. We therefore concluded that our recombinant hCD81 was most likely to be correctly folded and inserted into the *P. pastoris* membrane.

### Solubility screening and purification yields 1.75 mg/L β-OG-solubilized hCD81

**Fig. 4:**
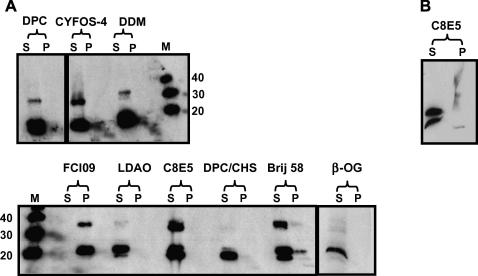
Solubility screen of hCD81 extracted from *P. pastoris* membranes. Both the micelle-bound solubilized proteins in the supernatant (S) and the non-solubilized proteins in the membrane pellet (P) were detected with an anti-hCD81 monoclonal antibody. Lane 1: *n*-dodecylphosphocholine (DPC), lane 2: cyclofos-4 (CYFOS-4), lane 3: *n*-dodecyl-β-d-maltopyranoside (DDM), lane 4: foscholineiso9 (FCI09), lane 5: lauryldimethylamine oxide (LDAO), lane 6: pentaethyleneglycol-*n*-octyl ether (C8E5), lane 7: *n*DPC-cholesterolhemisuccinate (DPC/CHS), lane 8: docosaethyleneglycolmonohexadecylether (Brij 58), lane 9: *n*-octyl-β-d-glucopyranoside (β-OG) (A). Repeat solubilization with C8E5. Both the micelle-bound solubilized proteins in the supernatant (S) and the non-solubilized proteins in the pellet (P) are shown (B). Samples were not exposed to any reducing agents. Molecular weights are in kDa.

A set of nine detergents and mixed micelles previously used and optimized for membrane proteins including PM28A/SoPIP2; 1 (detergent used: β-OG [25]), PagP barrel (CYFOS [26]), SERCA (DPC [27]), *Arabidopsis thaliana* leaf membrane proteins (Brij-*n* [28]) and five others were evaluated for effective hCD81 solubilization from *P. pastoris* membranes. Fig. 4 summarizes the solubilization of full-length hCD81 with these nine detergents where we examined the proportion of hCD81 solubilized into the supernatant compared with that remaining in the pellet in accordance with standard procedure [25]. The data show successful solubilization with eight of the nine detergents with some yielding soluble monomer (β-OG, DPC/CHS and LDAO) while others also apparently yielded various amounts of soluble dimer, as revealed by immunoblotting.

In order to investigate the possibility of dimer formation, hCD81 solubilized in C8E5 was prepared for analytical ultracentrifugation. It was clear from this experiment that dimer formation was an artifact of the analytical SDS–PAGE of this sample (Fig. 4B), as seen previously [29]. β-OG and DPC were therefore selected for subsequent solubilization and purification for biophysical and biochemical analysis because of the success reported throughout the literature with membrane proteins, and their suitability for downstream structural analysis.

**Fig. 5:**
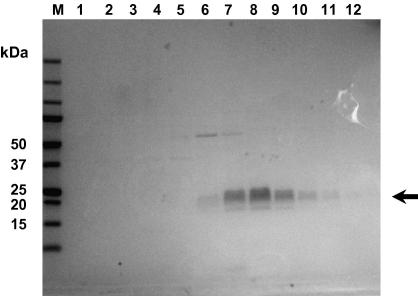
Coomassie-stained SDS–polyacrylamide gel showing the purification of recombinant hCD81 from *P. pastoris* membranes. Lane M: Marker, lane 1: wash 1 with buffer containing 2.5% β-OG, lane 2: wash 2 with buffer containing 2.5% β-OG, lanes 3–12: eluted fractions of hCD81 from the nickel affinity chromatography column using a linear imidazole gradient and buffer containing 1.0% β-OG. The arrow denotes hCD81. All samples were run in sample buffer containing β-mercaptoethanol.

Full-length hCD81 containing a carboxy-terminal His-tag and solubilized in β-OG was purified to near electrophoretic homogeneity by nickel-NTA agarose affinity chromatography. The protein eluted as a species with a molecular mass in the expected region of 26.6 kDa, at a yield of 1.75 mg/L (Fig. 5). Its identity was confirmed by mass spectrometry (data not shown). The higher molecular weight species seen in lanes 6 and 7 are most probably residual alcohol oxidase 1, as we have identified this host protein by mass spectrometry when producing other recombinant proteins in *P. pastoris*. The lower molecular weight form is likely to be due to residual imidazole or detergent present in the sample: the structural integrity under non-reducing conditions was confirmed by Western blotting with anti-hCD81 mAbs specific for correctly-folded, non-reduced disulfide-bridged protein (Fig. 2). Indeed, antibody binding in protoplasts, crude membrane fractions and purified hCD81 indicated the protein is intact throughout the purification protocol.

### Analytical ultracentrifugation indicates that recombinant hCD81 is a single species in detergent solution

**Fig. 6:**
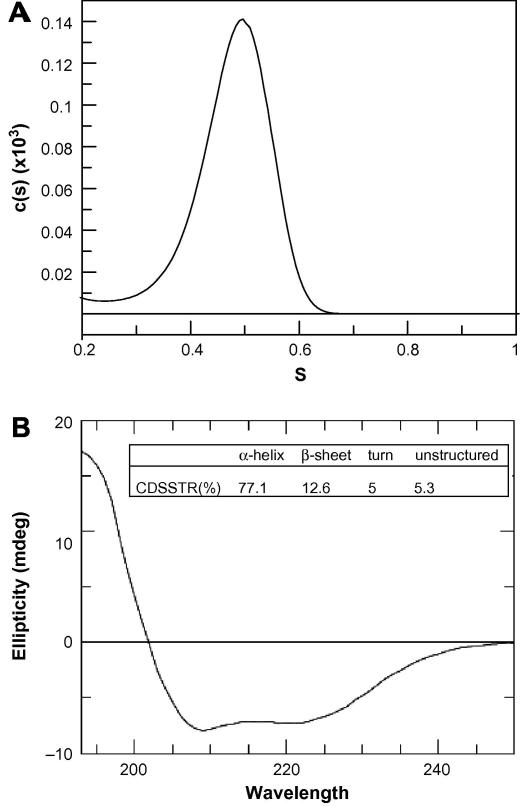
Analytical ultracentrifugation and CD data for hCD81 in β-OG micelles collected on a Proteome Lab XL-I instrument. Shown are the mass distributions of particles within the samples (A). Far-UV CD spectrum of DPC-solubilized hCD81 were recorded using a JASCO J-810 nitrogen-flushed spectropolarimeter. CD spectra were collected using a 0.2 cm path length cuvette and averaged over 8 scans in the far-UV domain. Spectra acquired were corrected for the buffer signal (B).

AUC data for β-OG-solubilized hCD81 revealed a single peak corresponding to 0.48 S and an absence of aggregated material, as shown in Fig. 6A. These data from AUC support the observation that dimeric species observed by SDS–PAGE may well be an artifact caused by the conditions used during the separation: no reducing agent was included in the loading buffer in order to maintain the conformation of the disulfide-bonded extracellular loop, EC2 (Figs. 2 and 4). The dimeric species do not appear on repeating the SDS–PAGE (Fig. 4B), and indeed have been similarly identified by others [29]. The peak observed by AUC is at a lower value compared with that expected for a 26.6 kDa protein. Transformation of the data to gain an estimate of mass leads to a predicted mass for the protein of 11.7 kDa. This anomalous behavior is likely to be a result of the interaction of β-OG with the protein. In Figs. 2, 4 and 5, the monomer also runs with a consistent, but somewhat lower molecular weight than 26.6 kDa (approximately 20 kDa in each case), which is not unexpected for membrane proteins [30].

### Circular dichroism indicates that recombinant hCD81 is predominantly α-helical in detergent solution

Measurement of circular dichroism spectra of DPC- and β-OG-solubilized hCD81 produced data consistent with a protein with a substantial α-helical component (Fig. 6B). These data were further deconvoluted by CDSSTR in order to produce a measure of secondary structure content for the protein (Fig. 6B), confirming the high α-helix content (77.1%, with a nRMSD for the fit of 0.091). This figure compares well with the α-helix content calculated from the known structural elements of hCD81. hCD81 contains four predicted transmembrane helices containing a total of 140 amino acids in an α-helical conformation, together with 83 amino acids in α-helices seen in the EC2 X-ray crystal structure [3]. This equates to a predicted helical content of 75.2% which is very close to that obtained from the CD analysis.

### Recombinant hCD81 interacts with HCV E2

**Fig. 7:**
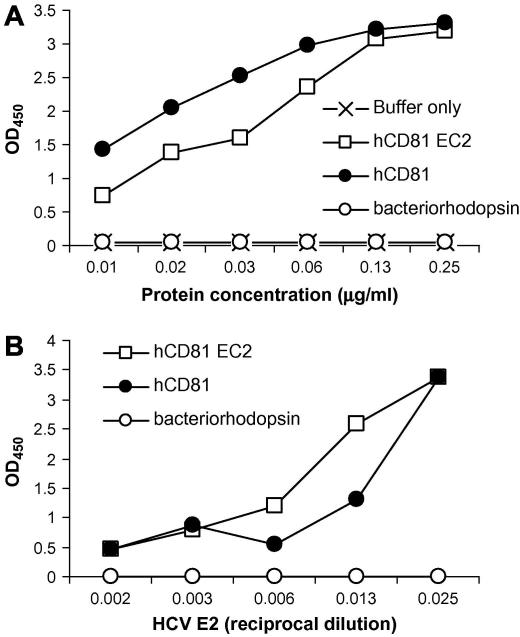
*Pichia pastoris*-produced CD81 binds Hepatitis C virus E2. Increasing concentrations of hCD81, bacteriorhodopsin and bacterially-produced hCD81 EC2 were bound to an ELISA plate and tested for reactivity with a saturating concentration of anti-CD81 M38. Data are expressed as optical density at 450 nm (A). A saturating concentration of *P. pastoris* hCD81, control bacteriorhodopsin protein and hCD81 EC2 were bound to an ELISA plate and tested for their ability to bind increasing amounts of soluble HCV E2. Bound E2 was realized with an anti-E2 mAb, 9/27 and anti-rat Ig-HRP. Data are expressed as optical density at 450 nm (B).

To investigate whether β-OG-solubilized hCD81 was able to interact with HCV E2 glycoprotein we established an ELISA to monitor interactions with a truncated form of E2 [21]. Increasing concentrations of hCD81 and a bacterially-produced form of CD81-EC2 [21] were allowed to bind to ELISA plates and their adsorption confirmed by following their reactivity with a saturating concentration of anti-hCD81 M38 (Fig. 7A). Full-length and truncated hCD81 bound M38 with similar profiles, confirming the antigenicity of the bound hCD81 antigen(s). Fig. 7B shows that HCV E2 bound full-length and CD81-EC2 with comparable efficiency.

## Discussion

We have demonstrated the first over-production of full-length, human CD81 at a yield of 1.75 mg/L of *P. pastoris* culture (Fig. 5). This yeast species allows ease of cultivation, optimization of production and access to the full complement of higher eukaryote-like post-translational modifications. This is in contrast to the widely-used prokaryotic microbe, *Escherichia coli*, in which accumulation of the recombinant protein in inclusion bodies requires refolding and can lead to much lower levels of fully-functional recombinant protein. To date several other recombinant mammalian membrane proteins have been produced in *P. pastoris* for example, mouse 5-hydroxytryptamine receptor (5HT_5A_) [31], human β2-adrenergic receptor [31], and bovine opsin [32]. Moreover, three of the five recombinant eukaryotic membrane proteins for which there are high-resolution structures in the protein data bank have been produced in *P. pastoris* [25,33,34].

In order to characterize purified hCD81, we used anti-hCD81 monoclonal antibodies that recognize epitopes within EC2 (Fig. 1). Recognition by these conformationally-specific antibodies suggested that the protein is correctly folded and allowed us to assess protein conformation by immunoblot and immunofluorescence from production through to purification. Immunoblotting revealed the integration of the native protein into the membrane of *P. pastoris* (Fig. 2, lanes 4) and this was further supported by results of the immunofluorescence and flow cytometric studies (Fig. 3) which revealed the majority of the protein located at the cell surface with little or no intracellular localization. The patchy distribution across the cell surface is reminiscent of other membrane proteins produced in yeast [35,36], and may be a result of differential segregation into different lipid raft domains due to changes in membrane potential, sterol composition and cell physiology [35,36].

Solubilization screening of hCD81 with nine different detergents, ranging from nonionic to zwitterionic molecules indicated solubilization in eight of the nine (Fig. 4). It was clear that the nonionic detergents such as *n*-octyl-β-d-glucopyranoside (β-OG), *n*-dodecylphosphocholine in the presence of cholesterol (DPC/CHS), and lauryldimethylamine oxide (LDAO) were far more successful in yielding soluble monomeric hCD81 than the zwitterionic cyclofos-4 (CYFOS-4) and foscholineiso9 (FCI09). Initial indications of dimeric species were shown to be artifacts of SDS–PAGE upon repeating the analysis (Fig. 4B). This phenomenon has also been observed for other tetraspanin-like proteins [29], and was due in part to the fact that no reducing agent was included in the loading buffer to maintain the conformation of the disulfide-bonded extracellular loop, EC2, in Figs. 2 and 4—note that reducing agent was present in the buffer used to prepare samples for Fig. 5. Based on these findings and other studies in the literature [25,27,37,38], β-OG and DPC were selected for solubilization scale-up and downstream processing of hCD81. Biophysical characterization of hCD81 in detergent solution employing AUC confirmed the presence of a single molecular weight species. Far-ultraviolet circular dichroism measurements produced a spectrum which predicts a high α-helical secondary structure that concurs well with that expected for hCD81. This purified protein was shown to bind HCV E2, suggesting that the protein adopts a conformation akin to the cell-surface-expressed molecule (Fig. 7).

To date no crystallographic analysis of full-length hCD81 has been performed due to the difficulties in obtaining sufficient amounts of stable, active, monomeric protein; a problem that besets the membrane protein field as a whole. However, an X-ray crystal structure of the soluble EC2 domain of hCD81 revealed five helices [3], for which loop structures were not clearly defined. The adjacent EC1 domain was absent [39], as was information on binding to any ligands or lipids. Moreover there are differences between the predicted three-dimensional model of the full-length hCD81 [40] and the EC2 structure [3]. The functional relevance of this soluble, 91 residue disulfide-bonded polypeptide structure is thus debatable, as is the dimer interface proposed from contacts seen in the crystal lattice. There is also significant evolutionary divergence in the protein sequences of ligand binding soluble domains among tetraspanins, thereby making extrapolation to other family members difficult.

The recent 6 Å cryo-EM structure of a naturally-occurring uroplakin tetraspanin 2D array (or “urothelial plaque”) revealed a rod-shaped structural morphology consisting of 4 TM helical bundles bound to a single TM helix partner [41]. However, differences between the proposed dimer and EC1–EC2 contacts with predicted models of CD81 [40], CD82 [42], and CD9 [43] exist and structural information is lacking about the critical intracellular carboxy-terminal domain which plays key roles in interaction with a variety of effector proteins [2,42]. Moreover the uroplakin tetraspanins are atypical members of the superfamily on account of their natural assembly into hexagonally-packed 2D crystals and their highly-specific interactions with a limited number of partner ligands. Together, this lack of structural information emphasizes the need for further structural and functional analysis of intact hCD81, itself a more typical tetraspanin with involvement in several key cellular processes. Questions include the role and positioning of EC1, the structure–activity relationships of the transmembrane domains in interactions with partner proteins and other tetraspanins, and specifically details of the mechanism of hCD81 signaling. Crucially, structural data on hCD81 will greatly facilitate site-directed mutagenesis studies to define individual amino acids that are critical for the interaction of the full-length molecule with HCV glycoproteins. Recent studies suggest important differences in the interaction of HCV with full-length, cell-expressed forms of hCD81 and recombinant forms comprising truncated CD81: some amino acid mutations exert a phenotype in the context of EC2 but not in the full length molecule [21]. Hence, analysis of interactions between full-length hCD81 and viral glycoproteins are required to investigate the critical amino acids defining this interaction [44].

To this end, we have produced full-length, recombinant hCD81 in a highly-pure, homogeneous, active form that is recognized by conformationally-specific antibodies, and which is predominantly α-helical in nature. This study represents the first biophysical characterization of a full-length recombinant tetraspanin, and opens the way for structure–activity analyses of this ubiquitous and important family of transmembrane proteins.

## Footnotes

2 *Abbreviations used:* EC1, extracellular loop 1; EC2, extracellular loop 2; hCD81, human CD81.
